# NR4A3 rearrangement reliably distinguishes between the clinicopathologically overlapping entities myoepithelial carcinoma of soft tissue and cellular extraskeletal myxoid chondrosarcoma

**DOI:** 10.1007/s00428-012-1240-0

**Published:** 2012-05-09

**Authors:** Uta Flucke, Bastiaan B. J. Tops, Marian A. J. Verdijk, Patricia J. H. van Cleef, Peter H. van Zwam, Pieter J. Slootweg, Judith V. M. G. Bovée, Robert G. Riedl, David H. Creytens, Albert J. H. Suurmeijer, Thomas Mentzel

**Affiliations:** 1 Department of Pathology, Radboud University Nijmegen Medical Center, P.O. Box 9101, 6500 Nijmegen, The Netherlands; 2 Department of Pathology, Leiden University Medical Center, Leiden, The Netherlands; 3 Department of Pathology, Maastricht University Medical Center, Maastricht, The Netherlands; 4 Department of Pathology, University Hospital Antwerp, Antwerp, Belgium; 5 Department of Pathology, University Medical Center Groningen, Groningen, The Netherlands; 6 Dermatopathologie Bodensee, Friedrichshafen, Germany

**Keywords:** Myoepithelial carcinoma of soft tissue, Extraskeletal myxoid chondrosarcoma, Soft tissue tumours, *EWSR1* rearrangement, *NR4A3* rearrangement

## Abstract

Myoepithelial carcinoma of soft tissue (MEC) and cellular extraskeletal myxoid chondrosarcoma (cEMC) share striking similarities. In this paper, we compare ten MECs with five cEMCs. MEC patients had an equal gender distribution. The age range was 15–76 years (mean, 42 years). Tumours were located on extremities, pelvic girdle, vulva and neck. Follow-up, available for nine patients, ranged from 4 to 85 months (mean, 35 months). Five patients were alive without evidence of disease, two were alive with disease and two died 8 months after the initial diagnosis. cEMCs were from three males and two females with an age range of 37–82 years (mean, 57 years); they presented in extremities, shoulder and paravertebral/cervical. Follow-up, available for four patients, ranged from 6 to 220 months (mean, 61 months). All patients were alive, two with recurrences and/or metastases and two without evidence of disease. Morphologically, the distinction between these two entities was difficult since all cases exhibited features typically seen in myoepithelial tumours. Immunohistochemically, MECs expressed pan-keratin (80 %), epithelial membrane antigen (EMA; 57 %), S100 (50 %), alpha-smooth muscle actin (ASMA; 75 %), calponin (67 %) and p63 (25 %). S100 and EMA were expressed in 40 % of cEMC cases respectively with additional immunoreactivity for p63, ASMA and glial fibrillary acidic protein in one case. Pan-keratin was negative in all neoplasms. *NR4A3* rearrangement was present in four of four cEMCs and in none of the MECs. In contrast, three of nine (33 %) MECs and four of five (80 %) cEMCs showed an *EWSR1* rearrangement. In summary, MECs and cEMCs share clinical, morphological, immunohistochemical and genetic characteristics. The pathognomic rearrangement of *NR4A3* is a useful diagnostic feature in identifying cEMCs.

## Introduction

Myoepithelial tumours of the soft tissue were initially characterised in a series by Kilpatrick et al. [1]. Later on, criteria for malignancy were established and the clinicopathological features expanded [2, 3]. Histologically, these tumours show the same broad variation in morphology as seen in their salivary gland counterparts [1–4].

Extraskeletal myxoid chondrosarcomas (EMCs) reveal a cord- or lace-like arrangement of small round to spindle-shaped cells with distinct eosinophilic cytoplasm distributed in a prominent myxoid stroma. In cellular lesions (cEMCs), amounting to one third of the cases, there is a greater morphological diversity [5–11]. Although the age range is broad for both entities, the peak incidence for EMC is the sixth and for MEC the fourth decade [2, 4, 12]. Furthermore, a significant subset of malignant myoepithelial tumours or myoepithelial carcinomas (MECs) occurs in children in contrast to EMCs which show a very low incidence in this cohort [3, 5, 8, 12, 13]. Both, myoepithelial tumours and EMCs arise predominantly in the proximal lower extremity but EMCs are more often located in the deep soft tissue [2, 4, 12]. Whereas EMCs have a protracted clinical course with a 10-year survival rate up to 88 % [5, 8, 14–16], MECs typically demonstrate aggressive behaviour, particularly in the pediatric population [2, 3]. Whether or not also cEMCs have a worse prognosis remains controversial [5–10].

Histologically, cEMC can be morphologically similar to myoepithelial tumours but the latter have a broader immunohistochemical profile [2–4, 12]. Gene fusions involving *NR4A3* (*nuclear receptor subfamily 4, group A, member 3*) located at 9q22 are characteristic for EMC and have never been described in myoepithelial tumours. In contrast, both tumour types harbour *EWSR1* (*Ewing sarcoma breakpoint region 1;* 22q12.2) rearrangement [3, 12, 17–25].

In this paper, we report on the morphological, immunohistochemical and molecular overlap of MEC and cEMC.

## Material and methods

We searched the surgical pathology and referral files of the authors for the diagnoses myoepithelial tumours of soft tissue and EMC. Slides were reviewed and the diagnoses were based on criteria according to the recent WHO classification [26]. Myoepithelial tumours with moderate to severe nuclear atypia (vesicular or coarse chromatin, prominent, often large nucleoli, or nuclear pleomorphism) and EMCs with cellular features were included in our study. Clinical details and follow-up were obtained from the referring pathologists (see “Acknowledgement”). Cases 8 and 10 were published earlier [3, 27, 28].

In all cases, the tissue was fixed in 4 % buffered formalin, routinely processed and embedded in paraffin; 2–4-μm-thick sections were stained with hematoxylin and eosin. Immunohistochemical methods consisted of the labelled Streptavidin Biotin technique using commercially available antibodies as listed in Table 1. Appropriate positive and negative controls were used throughout.

**Table 1:** Details of used immunohistochemical antibodies

Antibody	Clone	Dilution	Source
ASMA	1A4	1:500	DAKO, Glostrup, Denmark
EMA	Mc5	1:400	BioGenex, San Ramon, USA
CD34	HPCA-1	1:100	BD Biosciences, San Jose, USA
Pan-cytokeratin	MNF116	1:500	DAKO, Glostrup, Denmark
Pan-cytokeratin	AE1/3	1:50	DAKO, Glostrup, Denmark
S-100 protein	polyclonal	1:2000	DAKO, Glostrup, Denmark
P63	4A4	1:5000	Thermo Fisher Scientific, USA
GFAP	GA-5	1:200	DCS, Hamburg, Germany
Calponin	CALP	1:400	DAKO, Glostrup, Denmark

### Fluorescence in situ hybridisation analysis

Fluorescence in situ hybridisation (FISH) was performed as described earlier. For *EWSR1,* a directly FITC/Rhodamine-labelled break apart-probe (Abbott, Bergisch Gladbach, Germany) was used [29]. FISH probes to detect a *NR4A3* rearrangement (break apart probe) were generated in-house. BAC clones RP11-624K13 (centromeric), RP11-412F16 (centromeric), RP11-121L12 (telomeric) and RP11-467B11 (telomeric) were obtained from the BACPAC Resources Center (Oakland, CA). Clones were either labelled with biotin or digoxigenin using a nick translation kit, according to manufacturer’s instructions (Roche, Basel, Switzerland). Copy numbers of chromosome 9 were assessed using a centromere probe (CEP9). A negative control was used for each tumour. A case was considered having a break when at least 10 of 50 counted tumour cells (20 %) showed separation of a red and green signal.

### Reverse transcription polymerase chain reaction

RNA was isolated from formalin-fixed, paraffin-embedded material by proteinase K digestion, followed by phenol/chloroform extraction and *n*-propanol precipitation. cDNA synthesis was performed in a 24-μl reaction containing 1 μg of RNA, 1 μg of random hexamers (Promega) and 20 nmol dNTPs (Invitrogen) and heated at 65°C for 5 min. Next, 2 μl of RNasin (Promega), 8 μl of ×5 first-strand buffer (Invitrogen), 4 μl of 0.1 M DTT (Invitrogen) and 2 μl of Superscript II (Invitrogen) were added and the sample was heated accordingly: 20°C for 10 min, 42°C for 60 min and 95°C for 3 min.

For EMC, most potential translocation-specific *EWSR1-NR4A3* and *TAF15-NR4A3* fusion products were detected using primers targeting *EWSR1* (exon 7: TCCTACAGCCAAGCTCCAAGTC and exon 11: GACTCTAGATGATCTGGCAGAC, RefSeq: NM_005243.3), *TAF15 (TAF15 RNA polymerase II)* (exon 6: AGCAGTCAAATTATGATCAGCAGC, RefSeq: NM_003487.2) and *NR4A3* (exon 3: CCTGGAGGGGAAGGGCTATATTGGG, RefSeq: NM_006981.3).

## Results

### Clinical findings

Clinical details are summarised in Table 2. The MEC cohort consisted of five males and five females with an age range of 15–76 years (mean, 42 years). The tumours were located on the extremities (*n* = 4) with one case each on the thigh, calf, forearm and hand. Other sites were vulva (*n* = 2), gluteal (*n* = 1), sacral (*n* = 1) and the neck region (*n* = 1). In one case, the exact anatomic site was not known. Seven MECs were situated in the deep soft tissue and two subcutaneous. One superficially located tumour of the vulva showed exophytic growth. All patients underwent surgical treatment. Complete resection was reached in seven cases and marginal excision in one case. Tumour-positive margins were reported in two cases. Follow-up, available for nine patients, ranged from 4 to 85 months (mean, 35 months). Five patients were alive without evidence of disease at 12, 34, 39, 60 and 70 months, respectively. Two patients were alive with disease 4 and 85 months after the initial diagnosis, the latter with a second local recurrence. Two patients died of disease 8 months after the initial diagnosis. Case 10 was the first local recurrence on the hand with secondary bone involvement. Three patients presented with lung metastases. Involvement of regional lymph nodes was additionally observed in two of them, and one patient had bone metastases.

**Table 2:** Clinical data

Case No	Sex/Age	Primary Site	Size (cm)	Therapy	Follow-up (months)	Rec (Met)
MEC						
1	F/36 years	na/sc	4.5	R0	NA	
2	F/21 years	Thigh/deep	5.0	R0	70, NER	
3	F/53 years	Vulva/exophytic	3.5	R0	60, NER	
4	F/75 years	Vulva	3.5	R0	34, NER	
5	M/36 years	Forearm/deep	10	R0	8, DOD	Lung
6	F/15 years	Neck/deep	4	R2	85, AWD	2 rec
7	M/15 years	Sacral/deep	5	R0	39, NER	
8	M/17 years	Calf/deep	12	R0, perf,amp	8, DOD	Bone, lung, LN
9	M/71 years	Buttock/sc	15	R1	4, AWD	Lung, LN
10	M/76 years	Hand/deep, bone invasion	4.5	RM	12, NER	1 rec
EMC						
11	M/61 years	Thigh/deep	8.1	R0	12, NER	
12	M/58 years	Thigh/deep	10	R0	6, NER	
13	F/37 years	Shoulder/sc, deep	15	NT	6, AWD	LN
14	M/82 years	Paravertebral/cervical	NA	RX	NA	
15	F/46 years	Ankle	1	RM	220, AWD	4 rec, lung

*Mos* months, *Rec* recurrence, *Met* metastases, *Sc* subcutis, *LN* lymph node, *NER* no evidence of recurrence, *AWD* alive with disease, *DOD* death of disease, *NA* not available, *NT* no treatment, *Perf* limb perfusion, *Amp* amputation, *R0* complete resection, *RM* marginal resection, *R1* resection with histological positive margins, *R2* resection with macroscopically positive margins, *RX* resection with unknown margins

The five cases of EMC were from three males and two females with an age range of 37–82 years (mean, 57 years). Two lesions were located in the deep soft tissue of the thigh and one in the subcutis and soft tissue of the shoulder. One tumour each arose at the ankle and the paravertebral/cervical region. All patients but one (*n* = 4) underwent resection. Tumour free resection margins were reported in three cases (wide margins in two cases and marginal resection in one case). In Case 14, the resection status was not known. Follow-up, available for four patients, ranged from 6 to 220 months (mean, 61 months). No patient died of the disease so far. Case 15 was the 4th recurrence of a tumour with primarily classical morphological features. This patient had also pulmonary spread. Another patient was known with regional lymph node involvement (Case 13).

### Pathological findings

Grossly, the neoplasms were described as white or yellowish nodules, solid, gelatinous and also cystic in appearance. Haemorrhage and necrosis was seen in some of the lesions. The size range for MECs was 3.5 to 15 cm (mean, 6.7 cm) and for EMCs 1 to 15 cm (mean, 8.5 cm). In one EMC, the size was unknown.

**Fig. 1:**
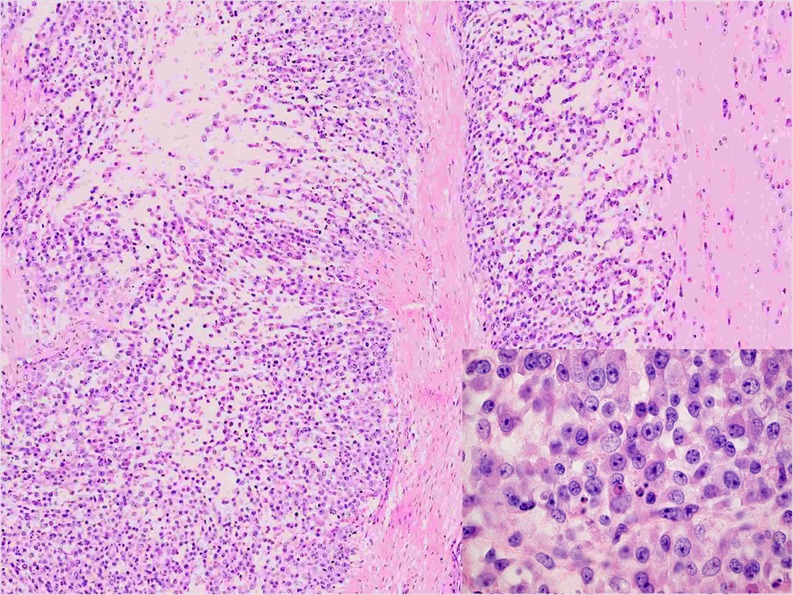
This case of MEC showed a reticular and cord-like pattern of epithelioid cells set in a prominent myxoid matrix (Case 5)

**Fig. 2:**
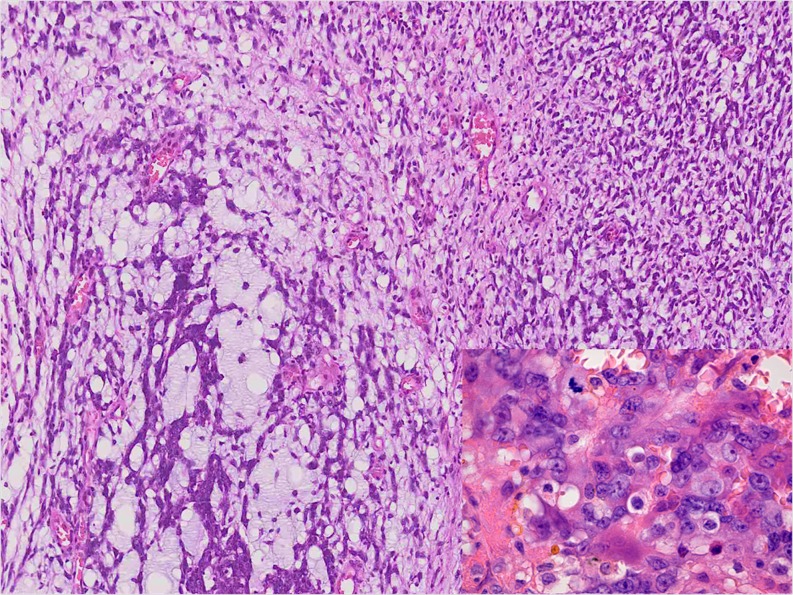
Note the nuclear pleomorphism, more often seen in MEC (Case 3)

Histologically, most cases demonstrated a (multi)nodular configuration with expansive margins and an incomplete pseudo-capsule. Infiltrative margins were focally seen. MECs showed in most of the cases varying growth patterns such as trabecular, reticular, nested and solid. A pure trabecular pattern was seen in two cases. Six tumours were composed of different cell types, including spindle, epithelioid, plasmacytoid and/or clear cells. Round cell morphology associated with clear cell features was observed in two cases and a pure epithelioid phenotype in two other cases (Fig. 1). Osteoclast-like giant cells were scattered in Cases 1 and 4. All cases exhibited moderate to severe nuclear atypia with vesicular or coarse chromatin (Fig. 2). Prominent nucleoli were conspicuous in seven cases. Mitoses ranged from one to six per ten HPF. The matrix was (chondro)myxoid in eight cases with pseudocystic changes in two cases. Five tumours showed stromal hyalinisation. Areas of haemorrhage and tumour necrosis were observed in one and two cases, respectively.

**Fig. 3:**
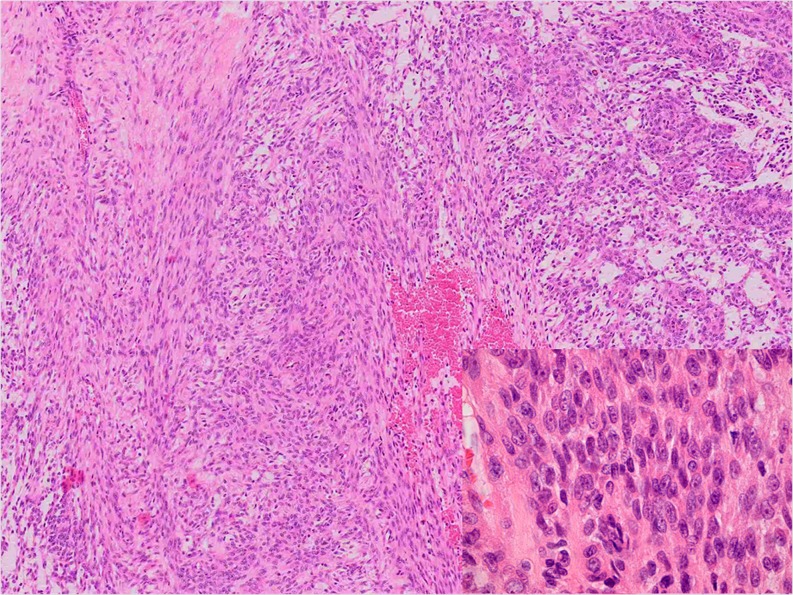
Monomorphic epithelioid and spindle-shaped cells in a cellular EMC (Case 11)

**Fig. 4:**
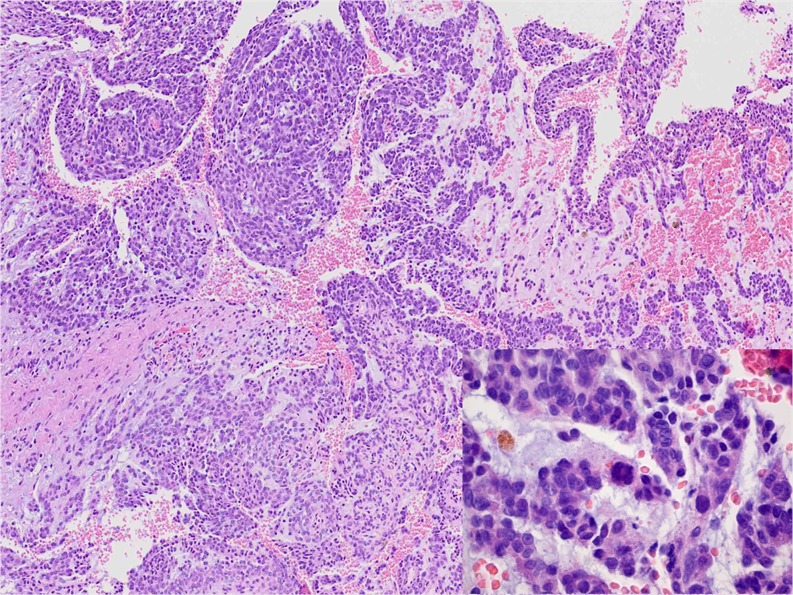
Solid and reticular arrangement of slightly polymorphic epitheloid cells in a cellular EMC

All EMC cases had focally classical features with strands and cords of small, uniform round to spindle-shaped cells set in a prominent myxoid matrix. There were round to oval nuclei and limited deeply eosinophilic cytoplasm. The tumour nodules, separated by fibrous septa, often showed peripheral cell condensation. In the cellular areas, sheets and nests of tumour cells were present. A trabecular and reticular-cystic pattern as well as loosely arranged cells occurred variably. The lesional cells were epithelioid and/or spindle shaped and slightly pleomorphic (Figs. 3 and 4). Case 13 showed increased pleomorphism, cytoplasmic vacuoles, multinucleated giant cells and prominent necrosis. In two cases, larger nuclei and prominent nucleoli were seen (Cases 11 and 12). Rhabdoid cytology was observed in Case 12. For all cases, the mitotic activity was very low and did not exceed one mitosis/ten HPF. In the cellular areas, the myxoid matrix was scant or even absent.

**Fig. 5:**
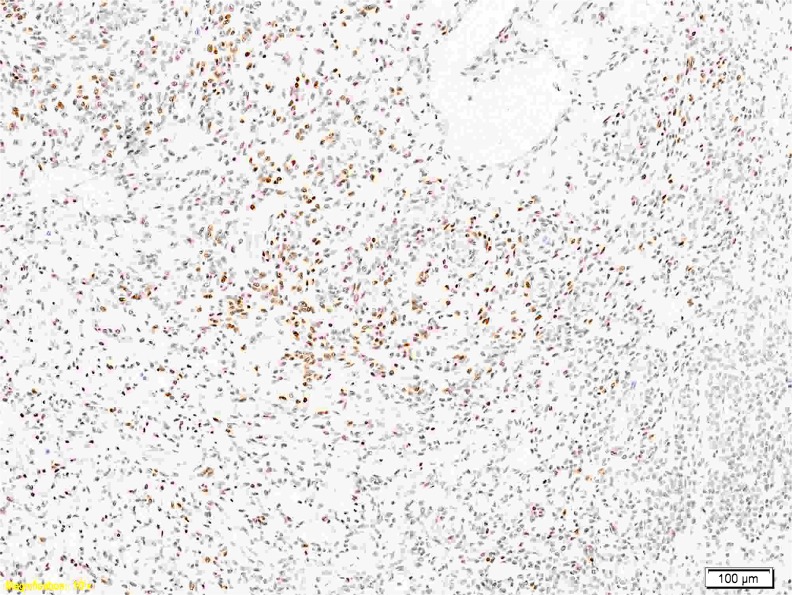
p63 was positive in one of the cellular EMC cases (Case 11)

Immunohistochemically, eight of ten (80 %) myoepithelial carcinomas were, at least focally, positive for pan-cytokeratin and four of seven (57 %) for epithelial membrane antigen (EMA). The two cases negative for pan-cytokeratin expressed EMA. Two of eight cases showed nuclear immunoreactivity for p63 (25 %). Alpha-smooth muscle actin (ASMA) was detected in six of eight (75 %) and calponin in four of six cases (67 %). A focal expression of S100 was seen in five of ten cases (50 %). Glial fibrillary acidic protein (GFAP), in four cases performed, was negative in all four. In cEMCs, S100 and EMA were each expressed in two of five cases (40 %). One of the mentioned S100-positive cases (Case 11) showed a broader pattern of marker expression with additional immunoreactivity for (Fig. 5) p63 and focally for ASMA and GFAP. Pan-cytokeratin was negative in all neoplasms (Table 3).

**Table 3:** Immunohistochemistry

Case	Pan-CK	EMA	p63	ASMA	S100	GFAP	Calponin
MEC							
1	−	+	−	f +	−	nd	+
2	+	nd	−	f +	f +	−	f +
3	f +	nd	−	f +	−	nd	f +
4	−	+	−	f +	−	−	nd
5	f +	−	nd	f+	−	nd	nd
6	+	nd	+	+	−	nd	nd
7	+	+	−	−	f +	−	−
8	+	−	nd	nd	+	−	+
9	+	−	−	−	f+	nd	−
10	+	+	+	nd	f+	nd	nd
	8/10	4/7	2/8	6/8	5/10	0/4	4/6
	80 %	57 %	25 %	75 %	50 %	0 %	67 %
EMC							
11	−	−	+	f +	f +	f+	nd
12	−	−	−	−	f +	−	nd
13	−	f+	nd	−	−	−	nd
14	−	−	−	−	−	−	nd
15	−	f +	−	−	−	−	nd
	0/5	2/5	1/4	1/5	2/5	1/5	
	0 %	40 %	25 %	20 %	40 %	20 %	

*f* focally, *nd* not done

### FISH and RT-PCR analysis

**Fig. 6:**
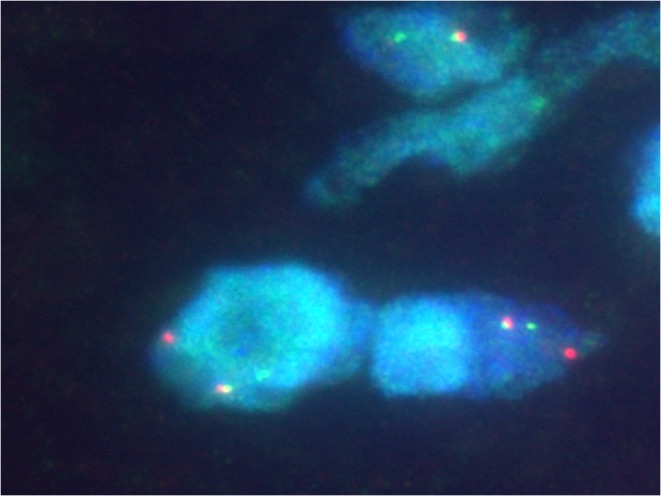
*NR4A3* rearrangement by FISH showing break-apart signal. This was observed in all EMC cases successfully tested

In three of nine (33 %) MECs, a *EWSR1* rearrangement was observed by FISH. One case failed for analysis due to poor hybridisation. No tumour showed a *NR4A3* rearrangement. Aberrations of chromosome 9 were seen in two instances. One of them harboured a polysomy and one a heterozygous deletion. All EMCs successfully tested (four of five) exhibited a *NR4A3* rearrangement (Fig. 6). One case failed for reverse transcription polymerase chain reaction (RT-PCR) and *NR4A3*-FISH. Three cases showed a *EWSR1* rearrangement by FISH. In one of them, a fusion of *TAF15-NR4A3* was additionally detected by RT-PCR. In Case 15, *NR4A3* was rearranged (by FISH) but a fusion with *EWSR1* or *TAF15* was not found (RT-PCR). *EWSR1-NR4A3* was evident in one neoplasm (Table 4).

**Table 4:** Molecular analyses (FISH*, RT-PCR)

Case	EWSR1*	*NR4A3*/cep 9*	TAF15-NR4A3	EWSR1-NR4A3
MEC				
1	+	−		
2	+	Heterozygote deletion chr. 9		
3	−	−		
4	−	−		
5	−	nd	−	−
6	+	−		
7	−	−		
8	−	Polysomy chr. 9		
9	−	−		
10	×	×		
EMC				
11	+	nd	+	−
12	nd	nd	−	+
13	+	+	×	×
14	+	×	×	×
15	nd	+	−	−

“+” rearrangement, “−“ no rearrangement, “×” analysis failed, *nd* not done

## Discussion

The first myoepithelial tumour of the soft tissue was published by Stout and Gorman in 1959 in a series of cutaneous lesions [30]. In the largest series to date, moderate to severe atypia (vesicular or coarse chromatin, prominent, often large nucleoli or nuclear pleomorphism) was determined as indicating malignancy [2]. The wide morphological and immunohistochemical diversity, presumably a result of the plasticity of myoepithelial cells, is the cause of the many differential diagnoses, which include most importantly the cellular variant of extraskeletal myxoid chondrosarcoma, furthermore atypical/malignant ossifying fibromyxoid tumour, undifferentiated carcinoma, epithelioid MPNST and proximal type epithelioid sarcoma. In cases with round cell morphology, Ewing sarcoma, myxoid/round cell liposarcoma and poorly differentiated synovial sarcoma are to be considered, at least in small samples [2–4].

The occurrence of myoepithelial tumors at different sites possibly reflects an aberrant gene expression pattern during oncogenesis rather than an origin from a specific cell lineage [3]. This is supported by the evidence of *EWSR1* rearrangement in a subset of benign and malignant myoepithelial tumours of skin, soft tissue, bone and visceral locations (lung) [25, 31]. The hitherto identified fusion genes are *POU5F1*, *PBX1* and *ZNF444* [23–25]. Other more heterogenous genetic changes are also identified including recurrent aberrations of chromosome 9 [27, 32–34]. Recently, a *pleomorphic adenoma gene 1* (*PLAG1*) rearrangement was discovered in a subset of benign mixed tumours of skin and soft tissues (with well-formed ducts). It seems that this genetic abnormality excludes *EWSR1* rearrangement [35]. Whether malignant myoepithelial/mixed tumours possess similar features remains unresolved as yet.

When Enzinger designated extraskeletal myxoid chondrosarcoma as a distinct entity he already mentioned a resemblance with salivary gland type, mixed tumours of deep fascial region of thigh with reference to the report by Dutra in 1960 [5, 36]. Classical cases of EMC have a typical histomorphology with uniform small round to spindle-shaped cells with deeply eosinophilic cytoplasm. The cells are arranged in a delicate network set in a copious myxoid stroma. Cellular variants, representing approximately one third of the cases, show greater morphologic heterogeneity, often resembling myoepithelial tumours [2, 4, 5, 8, 10, 11, 25, 33, 37, 38].

Both tumour types share features such as lobular/multinodular architecture, variable amounts of myxoid stroma and a reticular growth pattern. The stromal component in cellular areas can be poor or even absent. The cells are variably epithelioid, round and spindle shaped. Rhabdoid cytology may also be present in both. Unlike myoepithelial carcinomas, moderate to severe nuclear atypia is much less common in cEMC and can therefore be a helpful discriminating sign. This was also a finding in our series. Ductular structures and metaplastic cartilage or bone, present in not more than 20 % of soft tissue myoepithelial tumours, are not a feature of EMC [2–8, 10, 38–41]. None of our MECs demonstrated one of these characteristics.

Immunohistochemically, soft tissue myoepithelial tumours usually show expression of keratins (90–95 %), EMA (60 %), S100 (85 %), GFAP (50 %), calponin (90 %), SMA (40 %) and p63 (40 %) [4, 42]. In contrast, EMCs are often focally, positive for S100. GFAP, EMA, ASMA and keratins are expressed in a minority of cases, mostly with a focal staining pattern [2, 4, 6–8, 10, 12, 33, 38–41]. In our series, pankeratin was the most distinctive marker expressed in 80 % of MECs but in none of the EMCs. Although our series is small, this result mirrors those by others [8]. p63, labelling myoepithelial cells at different sites, is positive in circa 40 % of MECs and can be exceptionally positive in EMCs as we found in one of our cases [42].

Loss of *SMARCB1/INI1* is also an overlapping feature of the described entities and has been demonstrated in a subset of cases. Whereas underlying genetic alterations (homozygous deletion and frameshift mutation) have been detected in some EMCs, genetic aberrations have not been investigated in myoepithelial carcinomas as yet [3, 39]. Interestingly, the SMARCB1/INI1-negative EMC cases lack a typical major fusion gene transcript [39]. This raises the question whether these cases are more related to myoepithelial tumours. EMCs are defined by specific reciprocal translocations, involving the pathognomonic *NR4A3.* The described fusion partners are, in decreasing frequency, *EWSR1, TAF15, TCF12* and *TGF* [17–22].

By RT-PCR or FISH analysis, *NR4A3* rearrangement was found in all of our successfully tested EMCs. In one case, we detected besides a *TAF15-NR4A3* fusion an additional *EWSR1* rearrangement which is in line with another reported more complex rearranged case [43]. Furthermore, 33 % of our myoepithelial carcinomas showed *EWSR1* rearrangement, consistent with the results by Antonescu et al. [25]. We did not find *NR4A3* rearrangement in any of the myoepithelial carcinomas, but as previously reported, changes of chromosome 9 as a recurrent aberration were seen in two of our cases [27, 34].

In summary, myoepithelial carcinoma of soft tissue and cellular extraskeletal myxoid chondrosarcoma share clinical, morphological, immunohistochemical and genetic similarities. The pathognomic rearrangement of *NR4A3* and the general lack of keratin expression identify most cases of cEMC. The seemingly poorer outcome especially in young patients with myoepithelial carcinomas and possible different treatment options make discrimination between these different entities necessary.

## References

[1] Kilpatrick SE, Hitchcock MG, Kraus MD, Calonje E, Fletcher CDM (1997). Mixed tumors and myoepitheliomas of soft tissue: a clinicopathologic study of 19 cases with a unifying concept. Am J Surg Pathol.

[2] Hornick JL, Fletcher CDM (2003). Myoepithelial tumors of soft tissue. A clinicopathologic and immunohistochemical study of 101 cases with evaluation of prognostic parameters. Am J Surg Pathol.

[3] Gleason BC, Fletcher CDM (2007). Myoepithelial carcinoma of soft tissue in children: an aggressive neoplasm analysed in a series of 29 cases. Am J Surg Pathol.

[4] Gleason BC, Hornick JL (2008). Myoepithelial tumours of skin and soft tissue: an update. Diagn Histopathol.

[5] Enzinger FM, Shiraki M (1972). Extraskeletal myxoid chondrosarcoma. An analysis of 34 cases. Hum Pathol.

[6] Antonescu CR, Argani P, Erlandson RA, Healey JH, Ladanyi M, Huvos AG (1998). Skeletal and extraskeletal myxoid chondrosarcoma: a comparative clinicopathologic, ultrastructural, and molecular study. Cancer.

[7] Lucas DR, Fletcher CDM, Adsay NV, Zalupski MM (1999). High grade extraskeletal myxoid chondrosarcoma: a high-grade epithelioid malignancy. Histopathology.

[8] Meis-Kindblom JM, Bergh P, Gunterberg B, Kindblom LG (1999). Extraskeletal myxoid chondrosarcoma: a reappraisal of its morphologic spectrum and prognostic factors based on 117 cases. Am J Surg Pathol.

[9] Oliveira AM, Sebo TJ, McGrory JE, Gaffey TA, Rock MG, Nascimento AG (2000). Extraskeletal myxoid chondrosarcoma: a clinicopathologic, immunohistocheicl, and ploidy analysis of 23 cases. Mod Pathol.

[10] Okamoto S, Hisaoka M, Ishida T, Imamura T, Kanda H, Shimajiri S, Hashimoto H (2001). Extraskeletal myxoid chondrosarcoma: a clinicopathologic, immunohistochemical, and molecular analysis of 18 cases. Hum Pathol.

[11] Noguchi H, Mitsuhashi T, Seki K, Tochigi N, Tsuji M, Shimoda T, Hasegawa T (2010). Fluorescence in situ hybridization analysis of extraskeletal myxoid chondrosarcomas using EWSR1 and NR4A3 probes. Hum Pathol.

[12] Hisaoka M, Hashimoto H (2005). Extraskeletal myxoid chondrosarcoma: updated clinicopathological and molecular genetic characteristics. Pathol Int.

[13] Hachitanda Y, Tsuneyoshi M, Daimaru Y, Enjoji M, Nakagawara A, Ikeda K, Sueishi K (1988). Extraskeletal myxoid chondrosarcoma in young children. Cancer.

[14] Saleh G, Evans HL, Ro JY, Ayala AG (1992). Extraskeletal myxoid chondrosarcoma. A clinicopathologic study of ten patients with long-term follow-up. Cancer.

[15] Kawaguchi S, Wada T, Nagoya S, Ikeda T, Isu K, Yamashiro K, Kawai A, Ishii T, Araki N, Myoui A, Matsumoto S, Umeda T, Yoshikawa H, Hasegawa T (2003). Extraskeletal myxoid chondrosarcoma: a multi-institutional study of 42 cases in Japan. Cancer.

[16] Drilon AD, Popat S, Bhuchar G, D’Adamo DR, Keohan ML, Fisher C, Antonescu CR, Singer S, Brennan MF, Judson I, Maki RG (2008). Extraskeletal myxoid chondrosarcoma. A retrospective review from 2 referral centers emphasizing long-term outcomes with surgery and chemotherapy. Cancer.

[17] Sciot R, Dal Cin P, Fletcher C, Samson I, Smith M, de Vos R, Van Damme B, Van den Berghe H (1995). t(9;22) (q22-31;q11-12) is a consistent marker of extraskeletal myxoid chondrosarcoma: evaluation of three cases. Mod Pathol.

[18] Bjerkehagen B, Dietrich C, Reed W, Micci F, Saeter G, Berner A, Nesland JM, Heim S (1999). Extraskeletal myxoid chondrosarcoma: multimodal diagnosis and identification of a new cytogenetic subgroup characterized by t(9;17)(q22;q11). Virchows Arch.

[19] Sjögren H, Meis-Kindblom J, Kindblom LG, Aman P, Stenman G (1999). Fusion of the *EWS*-related gene *TAF2N* to *TEC* in extraskeletal myxoid chondrosarcoma. Cancer Res.

[20] Sjögren H, Wedell B, Meis-Kindblom JM, Kindblom LG, Stenman G (2000). Fusion of the NH2-terminal domain of the basic helix-loop-helix protein TCF12 to TEC in extraskeletal myxoid chondrosarcoma with translocation t(9;15)(q22;q21). Cancer Res.

[21] Panagopoulos I, Mertens F, Isaksson M, Domanski HA, Brosjö O, Heim S, Bjerkehagen B, Sciot R, Dal Cin P, Fletcher JA, Fletcher CD, Mandahl N (2002). Molecular genetic characterization of the *EWS/CHN* and *RBP56/CHN* fusion genes in extraskeletal myxoid chondrosarcoma. Genes Chromosom Cancer.

[22] Hisaoka M, Ishida T, Imamura T, Hashimoto H (2004). TFG is a novel fusion partner of NOR1 in extraskeletal myxoid chondrosarcoma. Genes Chromosom Cancer.

[23] Brandal P, Panagopoulos I, Bjerkehagen B, Gorunova L, Skjeldal S, Micci F, Heim S (2008). Detection of a t(1;22)(q23;q12) translocation leading to an EWSR1-PBX1 fusion gene in a myoepithelioma. Genes Chromosom Cancer.

[24] Brandal P, Panagopoulos I, Bjerkehagen B, Heim S (2009). t(19;22)(q13;12) Translocation leading to the novel fusion gene EWSR1-ZNF444 in soft tissue myoepithelial carcinoma. Genes Chromosom Cancer.

[25] Antonescu CR, Zhang L, Chang NE, Pawel BR, Travis W, Katabi N, Edelman M, Rosenberg AE, Nielsen GP, Dal Cin P, Fletcher CDM (2010). *EWSR1-POU5F1* fusion in soft tissue myoepithelial tumors. A molecular analysis of sixty-six cases, including soft tissue, bone and visceral lesions showing common involvement of EWSR1 gene rearrangement. Genes Chromosom Cancer.

[26] Fletcher CDM, Unni K, Mertens F (eds) World Health Organization Classification of Tumours. Pathology and genetics of tumours of soft tissue and bone. IARC Press, Lyon, pp 198–199, 213–215

[27] van den Berg E, Zorgdrager H, Hoekstra HJ, Suurmeijer AJH (2004). Cytogenetics of a soft tissue malignant myoepithelioma. Cancer Genet Cytogenet.

[28] Bell E, Van der Biezen JJ, Werker PM (2009). Parachordoma: a very rare tumor of the hand. J Hand Surg Eur.

[29] Hantschke M, Mentzel T, Rütten A, Palmedo G, Calonje E, Lazar AJ, Kutzner H (2010). Cutaneous clear cell sarcoma: a clinicopathologic, immunohistochemical, and molecular analysis of 12 cases emphasizing its distinction from dermal melanoma. Am J Surg Pathol.

[30] Stout AP, Gorman JG (1959). Mixed tumors of the skin of the salivary gland type. Cancer.

[31] Flucke U, Palmedo G, Blankenhorn N, Slootweg PJ, Kutzner H, Mentzel T (2011). *EWSR1* gene rearrangement occurs in a subset of cutaneous myoepithelial tumors: a study of 18 cases. Mod Pathol.

[32] Pauwels P, Dal Cin P, Roumen R, van den Berghe H, Sciot R (1999). Intramuscular mixed tumour with clonal chromosomal changes. Virchows Arch.

[33] Folpe AL, Agoff SN, Willis J, Weiss SW (1999). Parachordoma is immunohistochemically and cytogenetically distinct from axial chordoma and extraskeletal myxoid chondrosarcoma. Am J Surg Pathol.

[34] Hallor KH, Teixeira MR, Fletcher CDM, Bizarro S, Staaf J, Domanski HA, Vult von Steyern F, Panagopoulos I, Mandahl N, Mertens F (2008). Heterogeneous genetic profiles in soft tissue myoepitheliomas. Mod Pathol.

[35] Bahrami A, Dalton JD, Krane JF, Fletcher CDM (2011) A subset of cutaneous and soft tissue mixed tumors are genetically linked to their salivary gland counterpart. Genes Chromosom Cancer. doi:10.1002/gcc.20938 10.1002/gcc.20938 PMC3233652 22038920

[36] Dutra FR (1960). Mixed tumor, salivary gland type, of deep fascial region of thigh. Arch Path.

[37] Fletcher CDM, Fletcher CDM (2007). Soft tissue tumors. Diagnostic histopathology of tumors.

[38] Dei Tos AP, Wadden C, Fletcher CDM (1997). Extraskeletal myxoid chondrosarcoma: an immunohistochemical reappraisal of 39 cases. Appl Immunohistochem.

[39] Kohashi K, Oda Y, Yamamoto H, Tamiya S, Oshiro Y, Izumi T, Taguchi T, Tsuneyoshi M (2008). SMARCB1/INI1 protein expression in round cell soft tissue sarcomas associated with chromosomal translocation involving EWS: a special reference to SMARCB1/INI1 negative variant extraskeletal myxoid chondrosarcoma. Am J Surg Pathol.

[40] Oshiro Y, Shiratsuchi H, Tamiya S, Oda Y, Toyoshima S, Tsuneyoshi M (2000). Extraskeletal myxoid chondrosarcoma with rhabdoid features, with special reference to its aggressive behavior. Int J Surg Pathol.

[41] Abramovici LC, Steiner GC, Bonar F (1995). Myxoid chondrosarcoma of soft tissue and bone: a retrospective study of 11 cases. Hum Pathol.

[42] Jo VY, Fletcher CDM (2011). p63 immunohistochemical staining is limited in soft tissue tumors. Am J Clin Pathol.

[43] Turc-Carel C, Dal Cin P, Rao U, Karakousis C, Sandberg AA (1988). Recurrent breakpoints at 9q31 and 22q12.2 in extraskeletal myxoid chondrsarcoma. Cancer Genet Cytogenet.

